# Supplementary material for: MetaRibo-Seq measures translation in microbiomes
Source: Nat Commun. 2020 Jun 29;11:3268. doi: 10.1038/s41467-020-17081-z (PMC7324362; doi:10.1038/s41467-020-17081-z)
Supplement: Supplementary file 10 — Supplementary Data 7 [file 41467_2020_17081_MOESM10_ESM.zip › File2/Confidence_High_Taxonomy/226630_out.krona.html]

Javascript must be enabled to view this page.

members
magnitude
magnitudeUnassigned
count
unassigned
taxon
rank

226630\_out

19

superkingdom
19
2

1239
19
phylum

186801
19
class

186802
order
19

186803
19
family

841
genus
19


SRS018623\_contig\_number\_contig-100\_1617.82347SRS019161\_contig\_number\_contig-100\_1522.224379SRS019685\_contig\_number\_contig-100\_8176.127689SRS024075\_contig\_number\_contig-100\_20113.55923SRS024331\_contig\_number\_2154SRS043701\_contig\_number\_contig-100\_6583.50054SRS051031\_contig\_number\_36148SRS052027\_contig\_number\_16667SRS063040\_contig\_number\_contig-100\_497.245200SRS064645\_contig\_number\_contig-100\_2578.21705SRS065504\_contig\_number\_37135SRS077454\_contig\_number\_7150SRS077730\_contig\_number\_13298SRS097958\_contig\_number\_contig-100\_11926.11927SRS098644\_contig\_number\_contig-100\_16159.16159SRS098881\_contig\_number\_15376SRS147139\_contig\_number\_13444SRS148424\_contig\_number\_contig-100\_37536.91876SRS148817\_contig\_number\_contig-100\_6737.6738
360807
species
19
